# Idiopathic Eosinophilic Pleural Effusion Treated Successfully with Corticosteroid Therapy: A Clinical Case Report

**DOI:** 10.7759/cureus.3975

**Published:** 2019-01-29

**Authors:** Tirtha M Shrestha, Gaurav Nepal, Yow K Shing, Amir Joshi, Ravi R Pradhan

**Affiliations:** 1 Internal Medicine, Tribhuvan University Institute of Medicine, Kathmandu, NPL; 2 Internal Medicine, Yong Loo Lin School of Medicine, National University of Singapore, Singapore, SGP

**Keywords:** eosinophilic pleural effusion, eosinophilia, idiopathic, prednisolone

## Abstract

A pleural effusion is defined to be eosinophilic when 10% or more of the white blood cells in pleural fluid are eosinophils. Despite the multitude of studies enumerating the causes of eosinophilic pleural effusion (EPE), 14%-25% of such cases remain idiopathic even after thorough work-up. We report a case report of a 28-year-old never smoker male from the Rukum district of Nepal who presented to the emergency department (ED) with a chief complaint of shortness of breath associated with a low grade fever, nonproductive cough, and pleuritic right-sided chest pain for two weeks. There was no past medical and surgical history of note. Clinical examination revealed findings suggestive of a right-sided pleural effusion and relevant laboratory and radiological investigations were performed. Symptomatic treatment for the fever was administered. Full blood count showed a leukocytosis of 34 × 10^9^/L with an absolute eosinophil count (AEC) of 7.5 × 10^9^/L (22%). Peripheral blood smear showed normocytic normochromic erythrocytes with eosinophilia (morphologically normal eosinophils). Autoimmune profile was normal, inflammatory markers including erythrocyte sedimentation rate (ESR) and C-reactive protein (CRP) were raised, and an ultrasound and a plain radiograph of the chest confirmed the right-sided pleural effusion. Empirical anti-helminthic coverage was instituted. Subsequent infectious disease work-up was negative. An ultrasound-guided thoracentesis was performed, and the straw-colored pleural fluid showed an exudative picture which was eosinophil-predominant (42%). No malignant cells were detected. Failure of response to anti-helminthic therapy for one week led the team to start oral prednisolone 1 mg/kg once daily with the dose tapered subsequently. The patient responded dramatically. This was continued for one week and a regression of pleural effusion was demonstrated on chest radiography with a normalization of inflammatory parameters (ESR and CRP) and peripheral blood counts. Outpatient follow-up after one month showed no remaining clinical and radiological signs of EPE, and the peripheral eosinophilia resolved. As far as we know, this is the first case report of idiopathic EPE in the context of Asia. There are many causes of EPE, and some of them are still being discovered.

## Introduction

A pleural effusion is defined to be eosinophilic when 10% or more of the white blood cells are eosinophils [1]. Eosinophilic pleural effusion (EPE) has been attributed to many causes, of which the main broad categories include infectious, inflammatory, malignant, traumatic, and drug-related causes [2]. Despite the multitude of retrospective studies enumerating the causes of EPE, 14%-25% of such cases remain idiopathic even after thorough work-up [3].

The mechanisms leading to the recruitment of eosinophils to the pleural space have not been clearly established. Human and animal studies indicate that interleukin (IL)-5 is probably an important common contributor in the many possible pathogenetic pathways of EPE [4]. IL-5 helps in promoting eosinophil production, differentiation, activation, and inhibition of eosinophil apoptosis [5]. Additionally, IL-5 plays a role in stimulating eosinophil production of vascular endothelial growth factor (VEG-F), which increases vascular permeability and is thought to be a major mediator of the formation of exudative pleural effusions [6].

In working up EPE, it is imperative to look for a primary treatable cause, failing which, review of the drug intake, occupational and infectious disease exposure, comorbid conditions, pleural fluid re-analysis, additional imaging, and diagnostic thoracoscopy with pleural biopsy may be warranted as a search for less common diagnoses [7]. Idiopathic EPE is a diagnosis of exclusion. Patients with EPE without an obvious cause should be monitored until the effusion resolves or a known cause becomes apparent.

In this report, we present the case of a gentleman who presents to the hospital with a chief complaint of shortness of breath. After thorough evaluation, EPE was found to be the only cause of his symptoms, of which the etiology remained unknown. Whilst additional invasive investigations were afoot, a short course of prednisolone was started for which he showed dramatic improvement.

## Case presentation

A 28-year-old never smoker male from the Rukum district of Nepal presented to the emergency department (ED) of Tribhuvan University Teaching Hospital (TUTH) with a chief complaint of shortness of breath associated with a low grade fever, nonproductive cough, and pleuritic right-sided chest pain for two weeks. The dyspnoea was exertional in nature, and the patient did not complain of any orthopnea, paroxysmal nocturnal dyspnea, or swelling of the lower extremities. The low-grade fever had the same onset as the dyspnoea, had a Tmax of 99.8°F, and was not associated with chills or rigors. There was no history of sputum production, hemoptysis, night sweats, weight loss, or anorexia. He denies any rash, joint pain, numbness, or weakness in the extremities.

The patient had no past medical history of note, no history of allergies, and had not taken any drugs, medications or traditional therapies recently. There was no history of trauma to the chest or recent cardiothoracic instrumentation. He has been a farmer for 10 years and has tended to cows and goats from a young age. There is no history of occupational or residential exposure to asbestos, and no new plant or food exposure in the last three months. He has not travelled out of the country in the past year, and has had not any raw food ingestion, particularly of crustaceans, in the past three months. He has no family history of malignancy or autoimmune problems.

On examination, the vitals showed that he was hemodynamically stable with a temperature of 98°F, a sphygmomanometric blood pressure of 110/70 mmHg, heart rate of 88 beats/minute, and a respiratory rate of 22/minute saturating well in ambient air (peripheral spO_2_ 97%, as measured by pulse oximetry). There was no associated lymphadenopathy. There was, however, decreased excursion of the right chest wall, diminished breath sounds, and a stony dull percussion note on the posterior right lower zone of the chest. There were no other adventitious sounds heard. Symptomatic treatment in the form of paracetamol for the fever was started and investigations were ordered.

**Figure 1:**
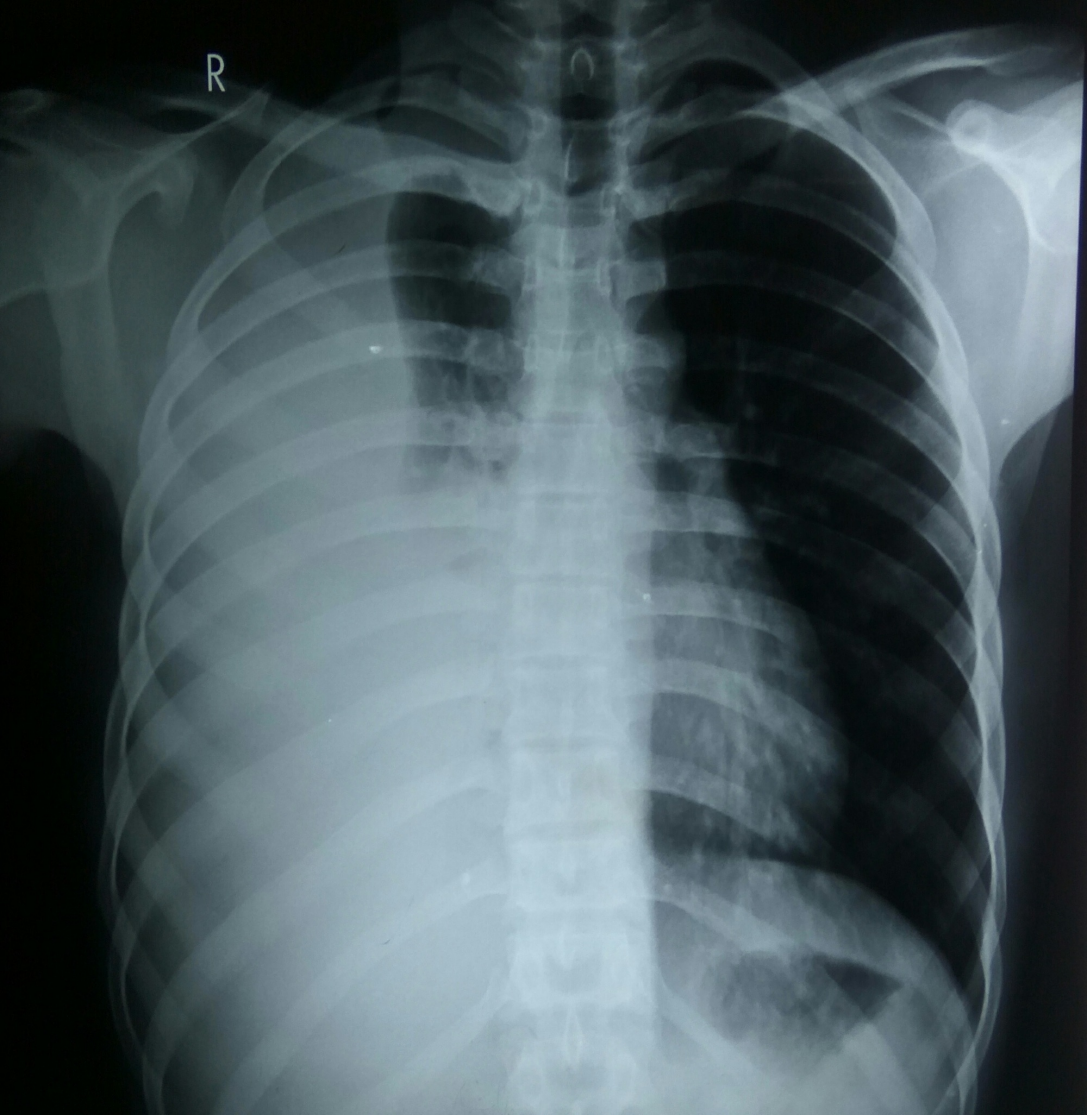
Posteroanterior erect chest X-ray of patient on initial presentation.

Full blood count showed a leukocytosis of 34 × 10^9^/L with an absolute eosinophil count (AEC) of 7.5 × 10^9^/L (22%). There was no concomitant derangement of the other cell lines (Hb 14.1g, platelet count 200 × 10^9^/L). Peripheral blood smear showed normocytic normochromic erythrocytes with eosinophilia (morphologically normal eosinophils). Serological examination was negative for antinuclear antibodies (ANA), anti-double stranded DNA antibodies (anti-dsDNA), rheumatoid factor (RF), anti-cyclic citrullinated peptide (CCP), and anti-neutrophil cytoplasmic antibodies (ANCA). Inflammatory markers including erythrocyte sedimentation rate (ESR) and C-reactive protein (CRP) were raised (40 mm/h and 72 mg/L, respectively). Ultrasonography revealed bilateral pleural effusion (right > left) with no free fluid or other gross abnormalities picked up in the abdominal and pelvic scan. Chest X-ray showed a right-sided pleural effusion, with no associated interstitial infiltrates, apical calcifications, hilar lymphadenopathy, or abnormalities in the pulmonary vasculature observed (see Figure 1).

As the primary team was concerned about an infectious etiology, anti-helminthic coverage in the form of a single-dose oral albendazole 400 mg was also administered. Subsequently, the team searched for epidemiologically relevant diseases such as tuberculosis, paragonimiasis, ascariasis, filariasis, and echinococcosis. Sputum smear was negative for mycobacterium and paragonimus ova. Serum adenosine deaminase was also normal (14.6 U/L). Additionally, the rest of the infectious disease work-up including sputum fungal stains and cultures, blood and urine cultures, stool ova and parasite work-up yielded no positive results.

In the interim, ultrasound-guided thoracentesis of the right chest wall was performed. Free fluid was noted in right pleural cavity; no loculations were identified and about 10 mL of straw-colored non-bloody fluid was aspirated and sent for cytological investigation. Pleural fluid was exudative with pleural fluid: serum protein ratio of >0.5, lactate dehydrogenase (LDH) ratio of >0.6, and pleural fluid LDH > ⅔ upper limit of normal for serum. Protein and glucose concentrations of the pleural fluid were measured to be 57.59 mg/dL and 4.9 mmol/L, respectively. Pleural fluid adenosine deaminase was also normal (23.1 U/L). Fungal and mycobacterial stains and cultures yielded no positive results. Malignant cells were not detected and the exudate was eosinophil-predominant (E 42%; N 40%; L 16%; M 2%).

**Figure 2:**
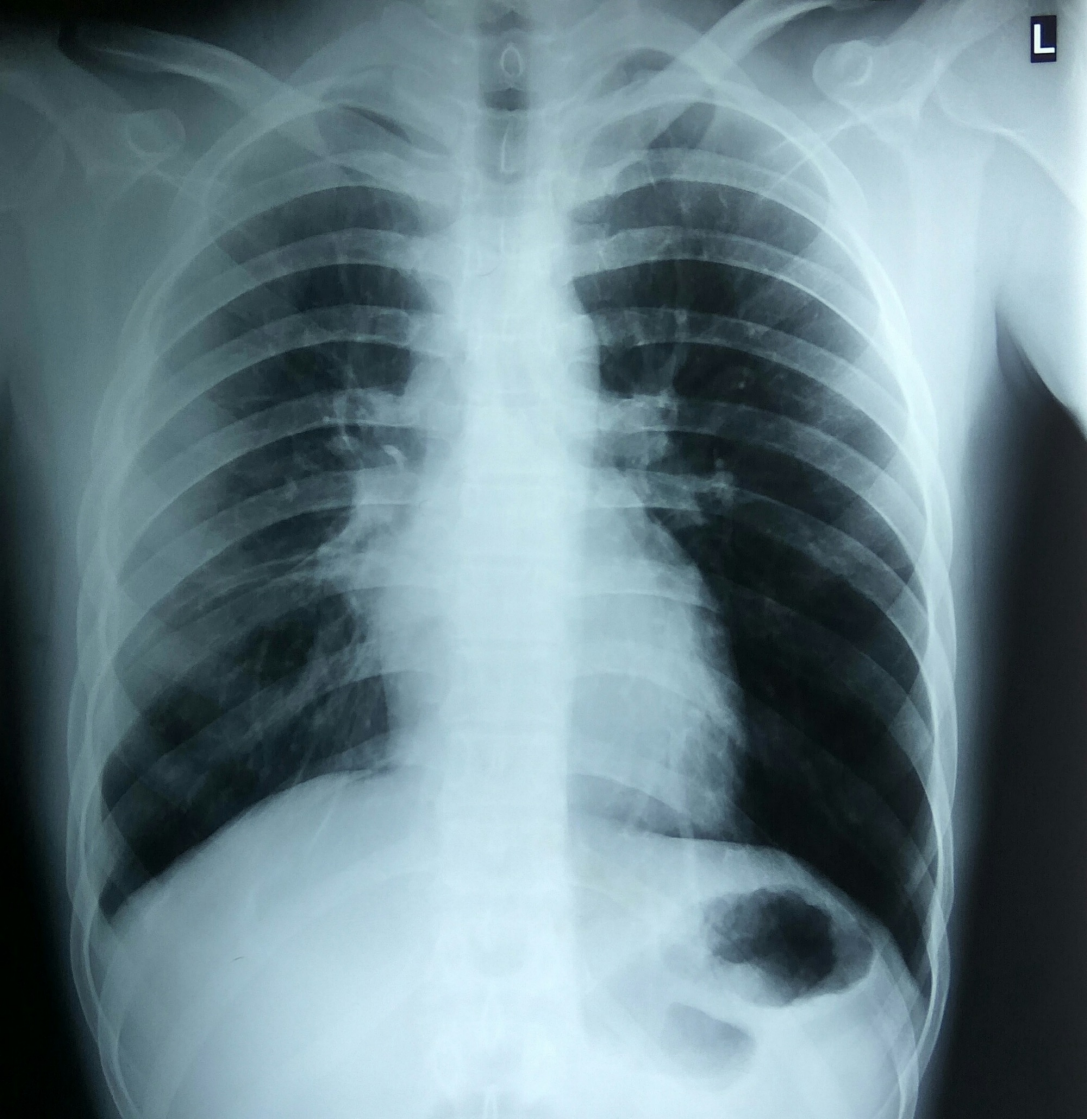
Posteroanterior erect chest X-ray of patient after one week of corticosteroid therapy.

Failure of response to anti-helminthic therapy for one week led the team to start oral prednisolone 1 mg/kg once daily with the dose tapered subsequently. The patient responded dramatically to prednisolone therapy. This was continued for one week and a regression of pleural effusion was demonstrated on chest radiography (see Figure 2) with a normalization of inflammatory parameters (ESR and CRP) and peripheral blood counts (AEC < 0.5 × 10^8^/L; N 86%; L 13%; Rest 1%). The patient was then discharged and advised for outpatient follow-up after one month and two months. During the follow-up consultation, the patient was well and there was no sign of pleural effusion clinically and radiographically. A repeat parasitological examination of the feces and sputum was conducted in the same setting. The test was negative, confirming the absence of the aforementioned suspected infections

## Discussion

Eosinophilic pleural effusion accounts for approximately 10% of all pleural effusions [2, 8]. This case report features a gentleman who presents with a right-sided pleural effusion found to be eosinophilic in nature. No primary cause was discovered on subsequent work-up and evaluation.

As a center based in Nepal, where tuberculosis is endemic with an incidence of 45,000 per annum [9], and where occasional cases of paragonimiasis are seen [10], one of the chief concerns was to screen and do an appropriate infectious disease work-up, especially in light of previous reports on tuberculosis accounting for 10% of EPEs in the Asian context [11] and case reports of paragonimiasis-associated EPE in the region [12-13]. The bevy of investigations in this regard, and for other parasitic infection, however, yielded no positive results.

Pleural trauma such as pneumothorax, haemothorax, thoracotomy, thoracoscopic surgery, or repeated thoracentesis has been reported to be associated with EPE. However, our patient had no history of pleural trauma and investigations for pleural trauma were negative [14]. Long-term asbestos exposure is also reported to be the cause of EPE. However, the history of our patient indicates that there is no such exposure [15]. Various drugs are related to the development of EPE, but the patient's medical history does not specifically indicate the cause of drug-related effusion. [16]. Other components of history and investigations did not particularly suggest a malignant effusion and effusion associated with rheumatological disease.

Recently, rare autoimmune cause of EPE has been reported in various parts of the world. Saito et al. previously wrote a case report on anti-PL-7 antisynthetase syndrome associated with EPE [17]. Our patient, however, was negative for ANA and dermatological findings (Gottron’s sign, mechanic hand sign, and shawl sign), effectively excluding this disease. Similarly, Mahgoub et al. also reported a case of immunoglobulin G4 disease associated with EPE [18]. However, the clinical and laboratory findings in that case was not similar to ours.

As the patient did not respond to anti-helmintic coverage and the cause of EPE was not established even after multitude of investigations, our team began a short course of steroids to improve the patient’s condition. Subsequently, the patient dramatically responded to therapy with prednisolone. This dramatic response corresponds to the diagnosis of idiopathic EPE. This was also observed in a Croatian case report of a 21-year-old female patient who had idiopathic EPE demonstrating a dramatic resolution of clinical symptoms and inflammatory markers with administration of 1 mg/kg oral methylprednisolone over the course of two weeks [3].

The patient in this report responded well to corticosteroid therapy. If a poor response was observed instead, the team would have performed a thoracoscopy along with a pleural biopsy to more effectively screen for malignancy and pleural tuberculosis. Bone marrow biopsy would also be considered for bone marrow diseases such as myeloproliferative neoplasms and hypereosinophilic syndrome.

## Conclusions

To the best of our knowledge, this is the first case report of an idiopathic EPE in the Asian context. There are many causes of EPE, and some are still being discovered till this day. We are only beginning to understand some of the pathophysiological mechanisms behind EPE and how these pathways manifest clinically.
